# The Book World of Medicine and Science

**Published:** 1896-11-07

**Authors:** 


### The Book World of Medicine and Science.

The Frog, an Introduction to Anatomy, Histology, and Embryology. By the late A. Milnes Marshall, M.D., F.R.S., &c. Sixth edition, revised and illustrated. Edited by G. Herbert Fowler, Ph.D. (London; David Nutt. 1896.)

An adequate knowledge of the anatomy, the minute structure, and the physiology of some animal other than man is a necessary preliminary to a medical student's training. It gives him an opportunity for noting resemblances and differences, and such comparison is the basis of all knowledge. From the ease with which it may be obtained in quantity, its convenience for laboratory work, and from the fact that it has been used by physiologists from time immemorial as a means of investigating the qualities of living tissues, the frog is probably the most suitable animal for the beginner, and every examination syllabus with which we are acquainted includes it. It is exceedingly satisfactory that so excellent a text-book as this of the late Prof. Milnes Marshall should be within the reach of every student. Dr. Fowler, the new editor, has had a long experience as a laboratory demonstrator, and the changes he has made in this edition will meet the approval of most teachers. He has made a number of minute alterations, which may seem trivial to a casual reader, but which are of practical importance. Such are the advice to distend the gullet with plugs of paper or cotton-wool before attempting to dissect the arteries, and directing students to shred, not to tease, striped muscular fibre. Among scientific additions, we note the inclusion of the adrenal bodies—structures which we make no doubt even many teachers would fail to point out correctly in the frog, and a proper description of the cartilaginous suspensorium of the jaw. The minuteness of the changes that have been made are an excellent testimony to the value of the former editions, and to the care and knowledge with which the present edition has been prepared.

The Drainage of Villages. By William Spinks, Assoc. M.Inst.C.E. (London: The Sanitary Publishing Company, Limited, 5, Fetter Lane, E.C. Price 1s.; cloth, 1s. 6d. net.)

This little monograph of some fifty pages, so the preface tells us, is founded on a lecture given a few years since in the West Riding of Yorkshire. The original matter has been expanded in legal and other directions into a short summary of useful information. But it bears very much the same relation to the subject under consideration as the popular scientific lecture does to the scientific text-book. In short, the book contains nothing that cannot be found in ordinary manuals of public health. At the same time it is only fair to state that Mr. Spinks has treated his subject, so far as he goes, well and accurately, and his little *brochure* will doubtless prove useful to general readers and persons to whom the more popular aspects of sanitation are of interest. If the title had simply been "Drainage," without any reference to villages, it would have been almost, if not quite, as descriptive of the general drift of contents.

Twentieth-Century Practice: An International Encyclopædia of Modern Medical Science. Edited by Thomas L. Stedman, M.D. Volume VI. Diseases of the Respiratory Organs. (London: Sampson Low, Marston, and Co. 1896).

This, the sixth volume of the "Twentieth-Century Practice of Medicine" fully comes up to the high standard attained by the parts already issued, and although we may be inclined to laugh at the somewhat pedantic aim at completeness which leads to the commencement of a volume on diseases of the respiratory organs with an article on acne of the nose, there can be no doubt that the subjects dealt with are very ably handled. The article by Dr. Prosser James on diseases of the nose enters temperately and judiciously into various more or less vexed questions relating to that organ. Dr. Moure, of Bordeaux, deals with diseases of the naso-pharynx and pharynx, and also with those of the tonsils. The section on diseases of the ear by Dr. Buck, of New York, is not altogether satisfactory, the operation procedures being alluded to rather than described. Diseases of the larynx are, on the other hand, very carefully and completely dealt with by Dr. Francke H. Bosworth, of New York, in a well illustrated article of over a hundred and sixty pages. This article, indeed, and the succeeding one on diseases of the trachea and bronchial tubes, by Sir Thomas Grainger Stewart and Dr. Gibson, taken together, form far the most important and interesting part of the volume before us. Dr. Winslow Anderson, of San Francisco, has satisfactorily treated of diseases of the lungs. Croupous pneumonia and tuberculosis, however, which, in practice, furnish so large a proportion of pulmonary cases, are dealt with elsewhere.

Deformities: A Treatise on Orthopædic Surgery. By A. H. Tubby, M.S.Lond., F.R.C.S.Eng. With 15 plates and 302 figures. (London: Macmillan and Co. 1896. Price 17s. net.)

This book must be read to be appreciated, although so well is it illustrated that a mere cursory turning of the pages tells how wide is the field it covers and how thoroughly the author has entered into the important problems which are involved in the subject of deformities. Commencing with deformities of the spine, caries is first dealt with, the characteristic deformity being well illustrated and described, as well as its relation to abscess and compression paraplegia. After a useful chapter on the physiology of the spinal column, the very large subject of scoliosis is entered upon, together with that of other deformities, such as lordosis and kyphosis, not the result of Pott's disease. In regard to scoliosis, it is shown that treatment must vary both according to the age of the patient and the stage which the curvature has reached; the first stage, or that in which the curves can be made entirely or almost to disappear by placing the patient in the improved position or by suspension or recumbency, being the only one in which real cure can be expected. The treatment which is recommended is a combination of exercises, recumbency, and correction by manipulation in the milder cases, while in fixed or severe cases supports must be used to relieve pain and pressure on viscera, and to prevent the deformity becoming worse. Some very striking illustrations are given of torticollis, the treatment of which condition is given under the three heads of manipulative, mechanical, and operative, preference being given in regard to operative procedures to the open method of dividing the sterno-mastoid. In the operative treatment of genu valgum preference is given to division of the shaft of the femur from the outside by means of the saw. There is a very complete and exhaustive account of the various forms of club foot, the description of the deformities being rendered very clear by admirable plates and woodcuts, and the directions for treatment being most carefully and definitely laid down. The work is well written, well printed, well bound, and most convincingly illustrated, and in every way redounds to the credit both of the writer and the publisher.

